# Characterization of tonsil infiltration and gene expression profile of innate sensors in PFAPA patients

**DOI:** 10.1186/1546-0096-9-S1-O30

**Published:** 2011-09-14

**Authors:** S Chiesa, MA Pelagatti, M Finetti, I Prigione, R Caorsi, D Lasiglié, R D’Agostino, A Sementa, A Omenetti, E Traggiai, A Martini, M Gattorno

**Affiliations:** 1 Laboratory of Immunology and Rheumatic Disease, U.O. Pediatria II, G.Gaslini Institute, Genoa, Italy, Department of Pediatrics, University of Genoa, Italy; 2 Laboratory of Oncology, G.Gaslini Institute, Genoa, Italy; 3 Unit of Otolaryngology, , G.Gaslini Institute, Genoa, Italy; 4 Service of Pathology, G.Gaslini Institute,Genoa, Italy

## Background

PFAPA syndrome is a common cause of periodic fever during early childhood belongs to the auto-inflammatory diseases. Palatine tonsils (PT) are sites where innate immunity leads to onset of the adaptive immunity, mediated by B and T lymphocytes. Three families of pathogen sensors mediate the recognition of microbes: Toll-like receptors (TLRs), NOD-like receptors (NLRs) and RIG-I-like receptors (RLRs).

## Aim

We aimed to investigate differences in leucocytes subpopulations and innate receptors gene expression of tonsils cells and peripheral blood (PB) from PFAPA patients to understand the pathogenesis of this disease.

## Methods

Tonsil tissue and PB were obtained from pediatric patients undergoing tonsillectomy: PFAPA patients (n=12) and control group, CG (n=15) .We performed staining of subpopulations on tonsils cells and tissues using flow cytometry and immunohistochemistry. We analyzed TLRs, NLRs, and RLRs gene expression by quantitative RT-PCR.

## Results

The histology of tonsils in PFAPA showed a preservation of tonsillar architecture without specific chronic inflammation with respect to CG. Preliminary results demonstrate an higher number of naïve CD4 and CD8 T cells and a significantly lower percentage of both effector memory T cells and functional Foxp3 T reg in PFAPA patients compared to CG. Then, we observed an increase NK cells in PFAPA patients with respect to CG. Tonsil cells expressed a broad repertoire of TLRs in PFAPA vs CG . The gene expression analysis of NLRs and RLRs receptors is ongoing.

## Conclusions

Results indicate a possible crucial role of NK cells and TLRs receptors during immune response in PFAPA patients. In addition, the high numbers of undifferentiated naïve T cells in PFAPA patients provide support for probable involvement of the adaptive immunity.

